# Application of comprehensive pharmaceutical care program in identifying and addressing drug-related problems in hospitalized patients with osteoporosis

**DOI:** 10.1186/s12913-022-08862-x

**Published:** 2022-11-28

**Authors:** Wenwen Chen, Houli Zhang, Juan Jiang, Xu Zhang, Jing Ding, Yanlin Liu, Heqin Dang

**Affiliations:** 1 Department of Pharmacy, the Second Affiliated Hospital of Shandong First Medical University, 366 Taishan Street, Tai’an, 271000 Shandong China; 2 Department of Stomatology, the Second Affiliated Hospital of Shandong First Medical University, 366 Taishan Street, Tai’an, 271000 Shandong China

**Keywords:** Drug-related problem, Osteoporosis, PCNE classification system, Clinical pharmacist, Comprehensive pharmaceutical care program

## Abstract

**Background:**

More information about the impacts of comprehensive pharmaceutical care program (CPCP) on the identification and resolution of drug-related problems (DRPs) is needed. This study aimed at researching the characteristics of DRPs in osteoporosis patients and evaluating the effect of CPCP in identifying and addressing DRPs.

**Methods:**

We performed a prospective interventional study in a teaching hospital. CPCP was established and conducted to identify and resolve DRPs by a multidisciplinary team (MDT) based on the Pharmaceutical Care Network Europe (PCNE) classification V9.0. Six pharmacists and one doctor worked directly in the study. All data was obtained from electronic medical records, direct observation and visits. The statistical analyses were performed using the SPSS Statistics software version 26.0.

**Results:**

Two hundred nineteen patients with osteoporosis were included in the final analysis. A total of 343 DRPs were identified, with an average of 1.57 DRPs per patient. The most common DRPs identified were “treatment safety P2” (66.8%; 229/343), followed by “other P3” (21.0%; 72/343) and “treatment effectiveness, P1” (12.2%; 42/343). The primary causes of DRPs were “dose selection C3” (35.9%; 211/588), followed by “drug use process C6” (28.9%; 170/588) and “drug selection C1” (12.6%; 74/588). Seven hundred eleven interventions were proposed to address the 343 DRPs, with an average of 2.1 interventions per DRP. The acceptance rate reached 95.9, and 91.0% of these accepted interventions were fully implemented. As a result, only 30 DRPs were unsolved before discharge. Additionally, the number of drugs was found to be associated with the number of DRPs significantly (*p* = 0.023).

**Conclusion:**

DRPs frequently occurred in hospitalized osteoporosis patients. CPCP could be an effect option to solve and reduce DRPs for osteoporosis patients and should be implemented widely to increase patient safety.

**Supplementary Information:**

The online version contains supplementary material available at 10.1186/s12913-022-08862-x.

## Background

Osteoporosis, characterized by reduction of bone mass and disruption of the microarchitectural structure of bone tissue, has developed into a serious public health concern worldwide [1]. Like other chronic medical conditions, hospitalized osteoporosis patients tended to be burdened with multiple comorbidities, particularly in postmenopausal women and elderly men [2]. Therefore, treatment of osteoporosis typically consisted of complicated medication regimens during hospitalization, which resulted in a high rate of drug-related problems (DRPs) [3–5]. Optimization of drug therapy to solve and prevent DRPs could ameliorate the healthy and economic burden for patients with osteoporosis [6, 7].

Currently, routine pharmaceutical services provided by pharmacists, such as prescription-checking, were hard to detect and address DRPs comprehensively [8, 9]. Therefore, it is necessary to establish comprehensive pharmaceutical care program (CPCP) to solve DRPs in patients with osteoporosis during the entire duration in the hospital. Additionally, the clinical characteristics of DRPs in patients with osteoporosis have varied greatly from study to study due to the lack of a normative classification system, making it difficult for comparison. In order to describe and assess the clinical characteristics of DRPs consistently, a standardized classification system of DRPs is crucial in hospital settings.

Of various classification systems that have been used to categorize DRPs in the world [10], the Pharmaceutical Care Network Europe (PCNE) classification system has been widely used in clinical practice and has internal consistency as it is updated and revised periodically [11]. Previous studies indicated that clinical pharmacists could record DRPs according to standard pattern with the help of PCNE classification system [12–15]. Up to now, there was only one study evaluating the prevalence and nature of DRPs in osteoporosis patients based on PCNE classification V6.2 10 years ago [16]. A better knowledge of DRPs among hospitalized osteoporosis patients based on the updated PCNE classification V9.0 would provide up-to-date and valuable information for medication management in the treatment of osteoporosis.

Therefore, it is of great significance to obtain sufficient data on the evaluation of CPCP and description of DRPs in patients with osteoporosis. This study represented an attempt to describe the characteristics of DRPs in hospitalized osteoporosis patients and the implementation of CPCP in identifying and addressing DRPs.

## Methods

### Study design, setting, and participants

This was a prospective interventional study conducted at the Second Affiliated Hospital of Shandong First Medical University. The hospital is a comprehensive teaching hospital with 1800 beds in the southern part of Shandong, a province in China.

Six pharmacists (two prescription-review pharmacists and four clinical pharmacists) and one doctor worked directly in the study.

All osteoporosis inpatients from May 2021 to December 2021 were included in the study. Inclusion criteria were defined as: (1) age ≥ 18 years, (2) diagnosed with osteoporosis, and (3) with an existing drug therapy on admission. Exclusion criteria were defined as: (1) patients had a terminal illness, or (2) could not consent to participate in the study.

The sample size was estimated with the Raosoft sample size calculator [17], which indicated a minimum of 197 patients were needed at 95% confidence level with a 5% margin of error (assigned 50% as the conservative assumption).

This study was approved by the Second Affiliated Hospital of Shandong First Medical University Ethics Committee (IRB number: K2020005). All patients provided written informed consent to participate in the study.

### Comprehensive pharmaceutical care program (CPCP)

**Fig. 1:**
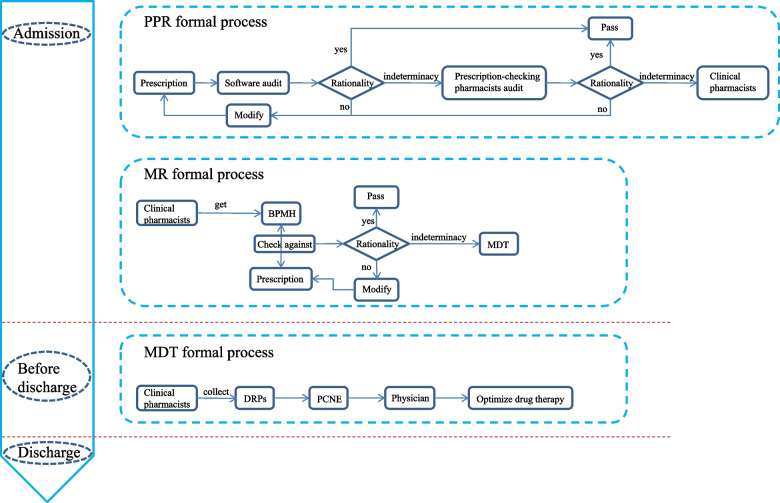
Flowchart of the implementation of CPCP. CPCP, comprehensive pharmaceutical care program; PPR, pre-prescription review; MR, medication reconciliation; BPMH, best possible medication history; MDT, multidisciplinary team; DRPs, drug-related problems; PCNE, Pharmaceutical Care Network Europe

In our hospital, CPCP included pre-prescription review (PPR) and medication reconciliation (MR) at admission, as well as multidisciplinary team (MDT) discussion before discharge. PPR was conducted by prescription-review pharmacists though a software. In PPR formal process, prescription was first audited by the software automatically based on pre-established medication rules, which called software audit. Unreasonable prescriptions were identified and fed back to physicians for modification. And then, the revised prescriptions would be automatically reviewed by the software again until approved. Notably, according to the updated drug label, clinical guidelines, expert consensus and actual drug use and management in the hospital, established medication rules would be modified continuously to improve accuracy of software audit. MR was provided by clinical pharmacists. In this process, clinical pharmacists got the best possible medication history (BPMH) including patients’ previous medication information through communicating with patients. And then, the doctor’s prescription was checked against BPMH to identify possible DRPs. In the process of PPR and MR, types and causes of identified DRPs were documented, along with clinical pharmacists’ interventions to solve the DRPs. Finally, clinical pharmacists would analyze all collected DRPs based on PCNE classification V9.0 and discuss the intervention results with physicians through MDT to optimize drug therapy. Flowchart of the implementation of CPCP in our hospital was shown in Fig. 1.

### Classification, identification and resolution of DRPs

All DRPs were categorized according to the PCNE classification V9.0, which included five parts: problems (P), causes (C), planned interventions (I), acceptance of the intervention proposals (A), and outcome of intervention (O). One problem may have multiple causes and lead to different interventions, but there is only one outcome eventually. All interventions were communicated to the doctors or patients. Interventions that required prescription modification were communicated to the doctors. While, interventions related to medication adherence were communicated to the patients. Accepted interventions were defined as the recommendations were agreed by physicians, and they were fully implemented, which resulted in a total resolution of DRPs. Two clinical pharmacists with more than 5 years’ experience independently identified and classified the DRPs. In case of doubt in the classification accuracy, a third clinical pharmacist with more than 15 years’ experience was consulted and a consensual decision was reached. An experienced physician was consulted about the medical knowledge associated with classification, identification and resolution of DRPs.

### Data collection

During the study, all data was obtained from electronic medical records, direct observation and visits. Patients’ demographics and clinical characteristics, patient diagnosis, history of active illness, comorbidities, family history, laboratory parameters, past medication history and daily medication list were collected at admission. The types and causes of identified DRPs were documented, along with clinical pharmacists’ interventions to solve the DRPs. Acceptance of interventions and status of DRPs were recorded 24 hours after interventions were proposed.

### Statistical analysis

Continuous data were presented as mean ± standard deviation if normally distributed or as median (interquartile range, IQR) if not normally distributed. Normality was assessed by the Kolmogorov-Smirnov test. Categorical variables were expressed as percentages. Both univariable logistic regression analysis and multivariable logistic regression analysis were conducted to determine the potential predictors of DRPs. The results of univariate and multivariate analysis were reported as crude odds ratio (COR) and adjusted odds ratio (AOR) at 95% confidence intervals (95% CI), respectively. A two-tailed *p*-value < 0.05 was considered to be statistically significant. The statistical analyses were performed using the SPSS Statistics 26.0 software (Chicago, IL, USA).

## Results

### Baseline characteristics

A total of 219 patients with osteoporosis were included in the final analysis. Patients’ mean age was 68.3 ± 8.5 years, and 32 patients (14.6%) were male. The majority (98.6%) of the patients were Chinese Han, and a total of 18 patients (8.2%) were drinkers. The most common comorbidities were hypertension (39.3%), followed by diabetes mellitus (25.1%) and coronary artery disease (24.2%). More than two-thirds (67.1%) of the patients were admitted to the orthopaedic ward. One hundred forty-seven patients were hospitalized for osteoporosis. The most commonly used osteoporosis medicine was Calcium Carbonate and Vitamin D3 Tablets (78.5%). The details of patients’ demographics and clinical characteristics were listed in Table 1.

**Table 1:** Patients’ demographics and clinical characteristics (*n* = 219)

Characteristics	Value
Gender, n (%)	
Male	32 (14.6)
Female	187 (85.4)
Age, mean ± SD, years	68.3 ± 8.5
Race, n (%)	
Chinese Han	216 (98.6)
Chinese Hui	3 (1.4)
BMI, mean ± SD	22.7 ± 4.4
T value, mean ± SD	−3.4 ± 0.9
Alcohol drinking, n (%)	18 (8.2)
Employment, n (%)	
Working	120 (54.8)
Not working	99 (45.2)
Previous Fracture, n (%)	29 (13.2)
Comorbidity, n (%)	
Hypertension	86 (39.3)
Diabetes mellitus	55 (25.1)
Coronary artery disease	53 (24.2)
Stroke	23 (10.5)
Rheumatoid arthritis	23 (10.5)
Admission ward, n (%)	
Department of orthopaedics	147 (67.1)
Department of rheumatology	29 (13.2)
Department of endocrinology	28 (12.8)
Department of breast	9 (4.1)
Others ^a^	6 (2.7)
Purpose of patient hospitalization, n (%)	
Osteoporosis-related	147 (67.1)
Not osteoporosis-related	72 (32.9)
Osteoporosis medicine, n (%)	
Calcium Carbonate and Vitamin D3 Tablets	172 (78.5)
Traditional Chinese Medicine	139 (63.5)
Risedronate Sodium Tablets	57 (26.0)
Calcitriol Soft Capsules	36 (16.4)
Alfacalcidol Tablets	20 (9.1)
Zoledronic acid Injection	15 (6.8)
Salmon Calcitonin Injection	14 (6.4)
Calcium gluconate injection	5 (2.3)

*BMI* Body Mass Index

^a^ 2 patients are in the chemotherapy ward, 2 patients are in the cardiology ward and 2 patients are in the psychiatric ward

### Prevalence and causes of DRPs

**Fig. 2:**
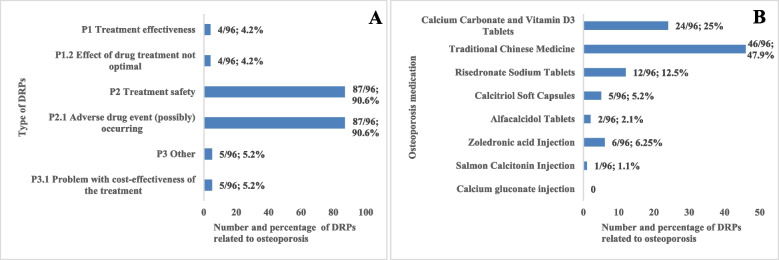
Number and percentage of DRPs related to osteoporosis according to type of DRPs (**A**) or osteoporosis medication (**B**)

A total of 343 DRPs were identified in 219 patients, with an average of 1.57 DRPs per patient. The most common DRPs were “treatment safety P2” (66.8%; 229/343), followed by “other, P3” (21.0%; 72/343) and “treatment effectiveness, P1” (12.2%; 42/343) (supplemental Table 1). Within the “treatment safety P2” domain, “Adverse drug event (possibly) occurring P2.1” was the only subcategory in this study. Additionally, 96 DRPs were directly related to osteoporosis treatment. Of which, the most common DRPs were “treatment safety P2” (90.6%; 87/96) (Fig. 2A). Notably, the osteoporosis medications behind DRPs were shown in Fig. 2B, and Traditional Chinese Medicine was most likely to cause DRPs (47.9%; 46/96).

As shown in supplemental Table 2, a total of 588 causes were identified for 343 DRPs. The primary causes of DRPs were “dose selection C3” (35.9%; 211/588), followed by “drug use process C6” (28.9%; 170/588) and “drug selection C1” (12.6%; 74/588). Within the “dose selection C3” domain, “dosage regimen too frequent C3.4” was the major subcategory (25.5%; 150/588), followed by “drug dose too high C3.2” (6.5%; 38/588), “dosage regimen not frequent enough C3.3” (3.1%; 18/588) and “drug dose too low C3.1” (0.9%; 5/588).

### Intervention and resolution of DRPs

Clinical pharmacists proposed 711 interventions to address the 343 DRPs, with an average of 2.1 interventions per DRP (supplemental Table 3). Most interventions were made at “prescriber level I1” (45.6%, 324/711), followed by “drug level I3” (39.0%; 277/711). As shown in Table 2, physicians accepted 95.9% (682/711) of the interventions, and 91.0% (312/343) of the DRPs were fully or partially solved (DRP status solved O1 and O2).

**Table 2:** Acceptance of interventions and the status of DRPs based on the PCNE classification V9.0

Primary domains	Code	Detailed classification	n (%)
*Implementation*			
Intervention accepted	A 1	***Total***	682 (95.9)
	A 1.1	Intervention accepted and fully implemented	634 (89.2)
	A 1.2	Intervention accepted, partially implemented	44 (6.2)
	A 1.3	Intervention accepted but not implemented	4 (0.6)
Intervention not accepted	A 2	***Total***	26 (3.7)
	A 2.2	Intervention not accepted: no agreement	1 (0.1)
	A 2.3	Intervention not accepted: other reason (specify)	4 (0.6)
	A 2.4	Intervention not accepted: unknown reason	21 (3.0)
Other	A 3	***Total***	3 (0.4)
	A 3.1	Intervention proposed, acceptance unknown	2 (0.3)
	A3.2	Intervention not proposed	1 (0.1)
*Outcome of intervention*			
Not known	O0	***Total***	1 (0.3)
	O0.1	Problem status unknown	1 (0.3)
Solved	O1	***Total***	284 (82.8)
	O1.1	Problem totally solved	284 (82.8)
Partially solved	O2	***Total***	28 (8.2)
	O2.1	Problem partially solved	28 (8.2)
Not solved	O3	***Total***	30 (8.7)
	O3.1	Problem not solved, lack of cooperation of patient	1 (0.3)
	O3.2	Problem not solved, lack of cooperation of prescriber	1 (0.3)
	O3.3	Problem not solved, intervention not effective	27 (7.9)
	O3.4	No need or possibility to solve problem	1 (0.3)

### Potential risk factors of DRPs

In univariable analysis, only one variable, number of drugs, had *p* value < 0.05. All variables were analyzed again in the multivariable binary logistic regression analysis (Table 3). Finally, the number of drugs was found to be the only risk factor of DRPs (AOR 1.046; 95% CI, 1.006-1.088; *p* = 0.023).

**Table 3:** Univariable and multivariable binary logistic regression analysis of predicators of DRPs

Variables	Univariable analysis		Multivariable analysis	
	COR (95% CI)	*p* value	AOR (95% CI)	*p* value
Age	1.011 (0.972-1.052)	0.574	1.011 (0.971-1.053)	0.607
Gender (female/male)	1.216 (0.487-3.036)	0.675	1.261 (0.494-3.218)	0.627
Number of drugs	1.045 (1.006-1.086)	0.023	1.046 (1.006-1.088)	0.023
Hypertension (yes/no)	0.940 (0.473-1.866)	0.859	0.917 (0.430-1.955)	0.823
Diabetes (yes/no)	0.802 (0.378-1.702)	0.566	0.649 (0.288-1.463)	0.297
Heart disease (yes/no)	1.756 (0.730-4.226)	0.209	1.808 (0.706-4.631)	0.217

## Discussion

To the best of our knowledge, this is the first prospective study to evaluate the prevalence of DRPs in patients with osteoporosis based on PCNE classification V9.0. DRPs were remarkably common in hospitalized patients with osteoporosis, with an average of 1.57 DRPs per patient.

The average number of DRPs in this study was higher than that in the previous study (0.39 DRPs per patient) conducted among Malaysian postmenopausal osteoporotic women prescribed bisphosphonates [16]. Discrepancies in the average number of DRPs might be explained by the following two reasons. First, the establishment and implementation of CPCP in the present study was very convenient for the identification of DRPs. With the help of pre-prescription review (PPR) system, DRPs could be identified automatically by software audit. However, counseling session was the only way to achieve DRPs in the previous Malaysia study. Second, different versions of PCNE classification system were used. This study used the PCNE classification V9.0 while the Malaysia study used the PCNE classification V6.2. These two versions were not compatible as many sections were added or revised in PCNE classification V9.0. For example, “possible adverse drug event occurring P2.1” was added into the PCNE classification V9.0, which indicated that many suspected adverse drug events would be identified as DRPs. Therefore, recent studies using similar version of PCNE reported similar incidence of DRPs as the present study [15, 18]. A research conducted in hospitalized patients with chronic obstructive pulmonary disease showed an average of 1.6 DRPs per patient [15]. Similarly, an average of 1.67 DRPs per patient was reported by Hon et al. in a study carried out in general paediatric ward [18]. All DRPs in the above two studies were categorized according to PCNE classification V8.02. Nevertheless，the average number of DRPs (1.9 DRPs per patient) presented in a prospective study conducted among geriatric patients was more than what was found in this study [19]. This could be explained by that geriatric patients were particularly vulnerable to DRPs caused by multiple factors such as polypharmacy and inappropriate prescribing [19].

In this study, more than half of the DRPs (66.8%) were related to treatment safety. The result was in line with previous studies, in which treatment safety was also the major type of DRPs [15, 16, 18, 20]. This indicated that clinical pharmacists should pay more attention to optimization of medication use to improve treatment safety. The primary cause of DRPs was “dose selection C3” (35.9%, 211/588). Namely, inappropriate dosage regimen and drug dose were major reasons for most DRPs in patients with osteoporosis. Considering this situation, CPCP conducted by clinical pharmacists was needed to play a unique role in addressing DRPs and ensuring the treatment safety for patients with osteoporosis. Clinical pharmacists performed clinical pharmacy activities through participating in clinical rounds and providing reasonable medication recommendations to support the MDT with a focus on identification, prevention, and resolution of DRPs to improve patient outcomes. Many studies had shown that clinical pharmacists played a crucial role in medication therapy management services in patients with osteoporosis by a multidisciplinary approach [21, 22]. For patients, medication education provided by clinical pharmacists was more conducive to solve the DRPs in the process of pharmacotherapy [23]. In our study, medication education was carried out frequently for osteoporosis patients during patient-pharmacist interview. Finally, a very high rate of intervention acceptance (95.9%) was reported in the present study, consistent with other related studies (91.0-99.4%) [15, 16, 19, 23]. As a result, 91.0% (312/343) of the DRPs were fully or partially solved (DRP status solved O1 and O2). This finding highlighted the importance of clinical pharmacists as part of the MDT, who could facilitate the identification of DRPs among patients and resolve the problems by providing reasonable medication recommendations for physicians or giving medication education to patients.

Consistent with findings from other studies [4, 24], this study also found that the number of drugs was the only predictor for DRPs occurrence. This could be explained by that as the number of drugs increased, there would be more possible drug-drug interactions and higher risk of medication errors, leading to more DRPs. Therefore, for patients with polypharmacy, clinical pharmacists should provide pharmaceutical care to identify and solve DRPs timely.

Several limitations should be mentioned. First, this was a single-center study with a relatively small sample size, which might prevent the generalization of our findings to other hospitals. Second, patients’ outcomes related to the resolution of DRPs were not available. Third, some risk factors of DRPs, such as number of diseases, number of review sessions and hospital stay days, were not researched in the present study.

## Conclusions

The prevalence of DRPs is relatively common in Chinese patients with osteoporosis. Implementation of CPCP could identify and solve DRPs effectively and play a positive role in optimizing medication therapy. Clinical pharmacists should pay special attention to osteoporosis patients with polypharmacy to identify and solve DRPs in a timely manner.

## Supplementary Information

**Additional file 1: Supplemental Table 1.** Primary domains of DRPs (total 343) based on the PCNE classification V9.0. **Supplemental Table 2.** Cause domains of DRPs (total 588) based on the PCNE classification V9.0. **Supplemental Table 3.** Proposed interventions (total 711) based on the PCNE classification V9.0.

## Data Availability

The data used to support the findings of this study are available from the corresponding author upon request.

## Abbreviations

CPCP: Comprehensive pharmaceutical care program
PPR: Pre-prescription review
MR: Medication reconciliation
BPMH: Best possible medication history
DRPs: Drug-related problems
MDT: Multidisciplinary team
PCNE: Pharmaceutical Care Network Europe
