# Supplementary material for: Application of comprehensive pharmaceutical care program in identifying and addressing drug-related problems in hospitalized patients with osteoporosis
Source: BMC Health Serv Res. 2022 Nov 28;22:1438. doi: 10.1186/s12913-022-08862-x (PMC9706996; doi:10.1186/s12913-022-08862-x)
Supplement: Supplementary file 1 — Additional file 1: Supplemental Table 1. Primary domains of DRPs (total 343) based on the PCNE classification V9.0. Supplemental Table 2. Cause domains of DRPs (total 588) based on the PCNE classification V9.0. Supplemental Table 3. Proposed interventions (total 711) based on the PCNE classification V9.0. [file 12913_2022_8862_MOESM1_ESM.docx]

**[Supplemental files]**

**Supplemental Table 1** Primary domains of DRPs (total 343) based on the PCNE classification V9.0

| Primary domains | Code | Detailed classification | n (%) |
| --- | --- | --- | --- |
| Treatment effectiveness | P1 | ***Total*** | 42 (12.2) |
|  | P1.2 | Effect of drug treatment not optimal | 39 (11.3) |
|  | P1.3 | Untreated symptoms or indication | 3 (0.9) |
| Treatment safety | P2 | ***Total*** | 229 (66.8) |
|  | P2.1 | Adverse drug event (possibly) occurring | 229 (66.8) |
| Other | P3 | ***Total*** | 72 (21.0) |
|  | P3.1 | Problem with cost-effectiveness of the treatment | 9 (2.6) |
|  | P3.2 | Unnecessary drug-treatment | 42 (12.3) |
|  | P3.3 | Unclear problem/complaint | 21 (6.1) |

**Supplemental Table 2** Cause domains of DRPs (total 588) based on the PCNE classification V9.0

| Primary domains | Code | Detailed classification | n (%) |
| --- | --- | --- | --- |
| Drug selection | C1 | ***Total*** | 74 (12.6) |
|  | C1.1 | Inappropriate drug according to guidelines/formulary | 3 (0.5) |
|  | C1.2 | Inappropriate drug (within guidelines but otherwise contraindicated) | 18 (3.1) |
|  | C1.3 | No indication for drug | 21 (3.6) |
|  | C1.4 | Inappropriate combination of drugs, or drugs and herbal medications, or drugs and dietary supplements | 16 (2.7) |
|  | C1.5 | Inappropriate duplication of therapeutic group or active ingredient | 10 (1.7) |
|  | C1.6 | No or incomplete drug treatment in spite of existing  indication | 1 (0.2) |
|  | C1.7 | Too many drugs prescribed for indication | 5 (0.9) |
| Dose selection | C3 | ***Total*** | 211 (35.9) |
|  | C3.1 | Drug dose too low | 5 (0.9) |
|  | C3.2 | Drug dose too high | 38 (6.5) |
|  | C3.3 | Dosage regimen not frequent enough | 18 (3.1) |
|  | C3.4 | Dosage regimen too frequent | 150 (25.5) |
| Treatment duration | C4 | ***Total*** | 35 (6.0) |
|  | C4.2 | Duration of treatment too long | 35 (6.0) |
| Dispensing | C5 | ***Total*** | 2 (0.2) |
|  | C5.2 | Necessary information not provided | 1 (0.3) |
|  | C5.3 | Wrong drug, strength or dosage advised (OTC) | 1 (0.3) |
| Drug use process | C6 | ***Total*** | 170 (28.9) |
|  | C6.1 | Inappropriate timing of administration or dosing intervals | 16 (2.7) |
|  | C6.2 | Drug under-administered | 13 (2.2) |
|  | C6.3 | Drug over-administered | 136 (23.1) |
|  | C6.5 | Wrong drug administered | 1 (0.2) |
|  | C6.6 | Drug administered via wrong route | 4 (0.7) |
| Patient related | C7 | ***Total*** | 65 (11.1) |
|  | C7.2 | Patient uses/takes more drug than prescribed | 48 (8.2) |
|  | C7.4 | Patient uses unnecessary drug | 5 (0.9) |
|  | C7.7 | Inappropriate timing or dosing intervals | 11 (1.9) |
|  | C7.8 | Patient administers/uses the drug in a wrong way | 1 (0.2) |
| Patient transfer  related | C8 | ***Total*** | 24 (4.1) |
|  | C8.1 | No medication reconciliation at patient transfer | 4 (0.7) |
|  | C8.2 | No updated medication list available | 1 (0.2) |
|  | C8.4 | Insufficient clinical information about the patient | 18 (3.1) |
|  | C8.5 | Patient has not received necessary medication at discharge from hospital or clinic | 1 (0.2) |
| Other | C9 | ***Total*** | 7 (1.2) |
|  | C9.1 | No or inappropriate outcome monitoring (incl. TDM) | 1 (0.2) |
|  | C9.2 | Other cause; specify | 3 (0.5) |
|  | C9.3 | No obvious cause | 3 (0.5) |

**Supplemental Table 3** Proposed interventions (total 711) based on the PCNE classification V9.0

| Primary domains | Code | Detailed classification | n (%) |
| --- | --- | --- | --- |
| No intervention | I0.1 | No intervention | 1 (0.1) |
| At prescriber level | I1 | ***Total*** | 324 (45.6) |
|  | I1.1 | Prescriber informed only | 132 (18.6) |
|  | I1.2 | Prescriber asked for information | 7 (1.0) |
|  | I1.3 | Intervention proposed to prescriber | 151 (21.2) |
|  | I1.4 | Intervention discussed with prescriber | 34 (4.8) |
| At patient level | I2 | ***Total*** | 100 (14.1) |
|  | I2.1 | Patient (drug) counselling | 4 (0.6) |
|  | I2.4 | Spoken to family member/caregiver | 96 (13.5) |
| At drug level | I3 | ***Total*** | 277 (39.0) |
|  | I3.1 | Drug changed to … | 4 (0.6) |
|  | I3.2 | Dosage changed to … | 46 (6.5) |
|  | I3.4 | Instructions for use changed to … | 150 (21.1) |
|  | I3.5 | Drug paused or stopped | 73 (10.3) |
|  | I3.6 | Drug started | 4 (0.6) |
| Other intervention or  activity | I4 | ***Total*** | 9 (1.3) |
|  | I4.1 | Other intervention (specify) | 1 (0.1) |
|  | I4.2 | Side effect reported to authorities | 8 (1.1) |
